# Burden and severity of deranged electrolytes and kidney function in children seen in a tertiary hospital in Kano, northern Nigeria

**DOI:** 10.1371/journal.pone.0283220

**Published:** 2023-03-17

**Authors:** Patience N. Obiagwu, Brenda Morrow, Mignon McCulloch, Andrew Argent

**Affiliations:** 1 Department of Paediatrics, Aminu Kano Teaching Hospital and Bayero University, Kano, Nigeria; 2 Department of Paediatrics and Child Health, Faculty of Health Sciences, University of Cape Town, Cape Town, South Africa; Foshan Sanshui District People’s Hospital, CHINA

## Abstract

**Introduction:**

Derangement in serum electrolytes and kidney function is often overlooked, especially in resource-constrained settings, and associated with increased risk of morbidity and mortality. This study aimed to describe the burden of derangements in serum electrolytes and kidney function in children presenting to a tertiary hospital in Nigeria.

**Methods:**

The laboratory records of all children who had serum electrolytes urea and creatinine ordered on their first presentation to hospital between January 1 and June 30, 2017 were retrospectively reviewed. Basic demographic data including admission status (inpatient or outpatient) were recordedandserum levels of sodium, potassium, chloride and bicarbonate were assessed for derangements usingnormal values from established reference ranges. Results of repeat samples were excluded. Kidney function was classified based on the serum creatinine relative to normal values for age and sex.

**Results:**

During the study period, 1909 children (60.3% male); median (IQR) age 42 (11.9) months had serum chemistry and 1248 (65.4%) were admitted. Results of their first samples were analyzed. Electrolyte derangements were present in 78.6% of the samples most commonly hyponatraemia (41.1%), low bicarbonate(37.2%), hypochloraemia (33.5%) and hypokalemia(18.9%). Azotaemia was found in 20.1% of the results. Elevated serum creatinine levels were found in 399 children (24.7%), 24.1% of those being in the severe category. Children aged 5 years and younger accounted for 76.4% of those with derangement in kidney function. One hundred and eight outpatients (17.8%) had deranged kidney function.

**Conclusion:**

Deranged serum electrolytes and kidney function were common in this cohort.

## Introduction

Many children who present to the emergency paediatric unit (EPU), paediatric intensive care unit (PICU) or outpatient department of any hospital have some imbalance in serum biochemistry [1, 2]. Electrolyte abnormalities are fairly common in children, more so in those who present to hospital with critical illnesses such as acute kidney injury [3]. While different authors have varying views on the frequency and need for routine testing for serum electrolytes and creatinine [4, 5], identification of abnormalities in serum electrolytes may impact on treatment and patient outcomes [2]. The use of clinical criteria for diagnosing electrolyte derangement and kidney disease may underestimate the burden, as clinical features indicative of kidney damage may appear only at an advanced stage of the disease, hence the associated high morbidity and mortality [6]. Kidney function in children is usually determined from the estimated glomerular filtration rate (eGFR) using either the original Schwartz formulaorthebedside Schwartz formula, which require input of both serum creatinine value and the height of the child [7, 8], despite the limitations of serum creatinine as a marker of kidney function [9]. Kidney dysfunction is defined and the severity categorized into stage 1 (mild), stage 2 (moderate) and stage 3 (severe) based on the degree of derangement in serum creatinine above known or estimated baseline values and/or if the volume of urine produced is available, the degree of reduction in urine output over defined periods of time.

Kidney failure is usually suspected when the serum creatinine level is greater than the upper limit of “normal” [10]. However, the production of creatinine during critical illness varies for several reasons [11] (e.g., serum creatinine is elevated in patients with significant catabolism, while serum creatinine is diluted by volume resuscitation). By the time serum creatinine starts to rise, a significant proportion of kidney function, about 50%, may already have been lost [12]. Small increments in serum creatinine above normal values have been associated with higher mortality [13].

Serum urea is also a marker of kidney function [10], and may raise pointers to common clinical conditions such as fever and dehydration, which could ultimately lead to kidney injury. In some resource-constrained settings, the prolonged turnaround time for the receipt of the serum chemistry results may render the results useless, leading to some healthcare providers not requesting serum chemistry and missing potential derangements. Furthermore, in certain centres, a system to flag abnormal results is not in place and thus the healthcare providers are not made aware of such results which may lead to preventable morbidity and mortality.

While studies have reported on the burden of deranged kidney function in hospitalized children, children with specific conditions andchildren seen at specialist clinics in health institutions [14–17], there is a paucity of studies describing the electrolyte abnormalities in children presenting with paediatric problems to Nigerian hospitals [18–20] and no studies from Kano, the most populated city in Nigeria. This study therefore aimed to describe the overall burden of electrolyte and kidney function derangement in children who had serum electrolytes, urea and creatinine ordered by their attending physician. The resource-limited characteristic of the region makes the study findings critical to patient management.

## Methods

This was a retrospective review of laboratory records of children who had serum chemistry testing in Aminu Kano Teaching Hospital (AKTH), a tertiary health center in north-western Nigeria, between January 1 and June 30, 2017. The study was approved by the Research Ethics Committee of AKTH and the Faculty of Health Sciences Human Research and Ethics Committee (FHS HREC), University of Cape Town. Being a retrospective review of laboratory results, the need for informed consent was waived. Basic sociodemographic data of the patients as well as the results of serum sodium, potassium, chloride, bicarbonate, urea and creatinine testing were extracted from laboratory records. Patients were classified into three age groups: less than 12 months (Infant), 12 months to 60 months (Under-five) and greater than 60 months (Older child). Patient sources were classified as inpatient (patients admitted to hospital wards) or outpatient (patients seen in clinics and outpatient departments). Each source was further categorized intomedical or surgical. Repeat samples on same patients as identified by documented name, age, sex and hospital number were excluded. Results with missing data for age, sex or sample source were also excluded. The serum chemistry records books used in the department of Chemical Pathology do not contain any clinical information or diagnoses of patients. Thus, there were no known or excluded clinical conditions.

The normal ranges used for the serum electrolytes, urea and creatinine, as well as terminologies used for deranged values are listed in the S1 Appendix. The upper limits of normal (ULN) for age and gender were used for classification of derangements in serum urea and creatinine [21].

Data were entered into an Excelspreadsheet(Microsoft Excel, version 2007) and exported to SPSS version 20.0 for statistical analysis. Categorical variables were expressed as proportions and associations determined using the Pearson Chi square test. Continuous variables were not normally distributed as determined visually using the histogram and the Q-Q plots and statistically using the Kolmogorov-Smirnov Z test. They were thus reported as medians with interquartile ranges (IQR) and mean ranks compared using the Mann-Whitney U test for two groups and Kruskal-Wallis test for more than two groups. A p-value of <0.05 was considered to be statistically significant.

### Inclusivity in global research

Additional information regarding the ethical, cultural, and scientific considerations specific to inclusivity in global research is included in the S1 File.

### Results

**Fig 1:**
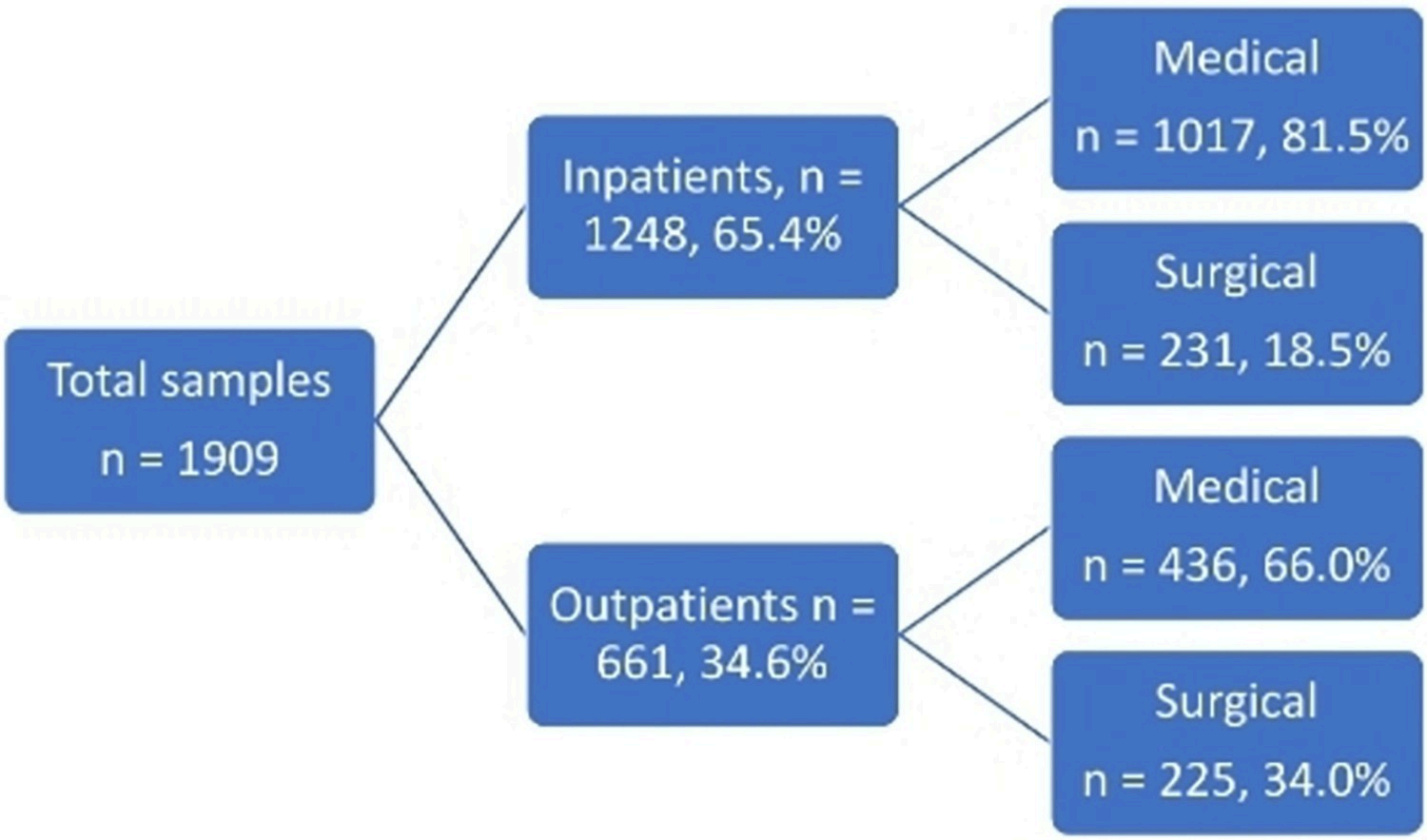
Distribution of sources of analyzed samples.

A total of 5574 serum chemistry tests were done on children during the study period, 2387 being repeat tests and 1278 results having incomplete information. Thus, the results of 1909 children [1152 (60.3%)male] were analyzed. Median (IQR) age was 42 (11,93) months with no significant age difference between males and females (p = 0.455). There were 1226 (64.2%) children under 5 years of age, with infants accounting for 42.4% of this proportion. Of 1248 (65.4%) admitted children, 1017 (81.5%) were medical admissions. There were reports available on 1908 (99.9%) serum sodium and chloride, 1896 (99.3%) serum potassium, and 1899 (99.5%) serum bicarbonate results. Serum urea results were available in 1905 children (99.8%) while 1618 children (84.8%) had their serum creatinine results available. Fig 1 shows the distribution of the sources of the samples while basic demographics and median (SD) serum chemistry values for the different age groups are shown in Table 1.

**Table 1:** Basic demographics and median (IQR) values of serum analytes for all age groups.

	Age group (months)				
**Parameter**	**< 12 months**	**12 to 60 months**	**>60 months**	**χ** ^**2**^	**P**
Total number (%)	520 (27.2)	706 (37.0)	683 (35.8)		
Male, n (%)	314 (60.4)	434 (61.5)	404 (59.2)		
Age, median (IQR)	3.0 (0.4, 7.0)	36.0 (24.0, 48.0)	108 (84.0, 132.0)		
Admitted, n (%)	414 (79.6)	437 (61.9)	397 (58.1)	66.211	0.000
**Analyte, median (IQR)**					
Sodium, mmol/L	137 (131, 141)	135 (130, 141)	136 (131, 142)	6.580	0.037
Potassium, mmol/L	4.3 (3.8, 5.0)	4.0 (3.6, 4.6)	3.9 (3.5, 4.4)	66.720	0.000
Chloride, mmol/L	98.0 (94.0, 103.0)	96.0 (93.0, 102.0)	97.0 (93.0, 103.0)	10.607	0.005
Bicarbonate, mmol/L	20.0 (18.0, 22.0)	21.0 (18.0, 23.0)	20.0 (19.0, 24.0)	40.171	0.000
Urea, mmol/L	3.8 (2.7, 7.0)	3.6 (2.6, 5.4)	3.7 (2.8, 5.5)	6.124	0.047
Creatinine, μmol/L	54 (38.0, 84.0)	49.0 (36.0, 68.0)	52.0 (40.0, 73.0)	19.655	0.000

IQR–interquartile range; χ^2^ = Chi square value for Kruskal Wallis test.

Admitted patients were younger, and with lower values of serum electrolytes and higher values of serum urea and creatinine when compared to those of the outpatients (Table 2).

**Table 2:** Comparative median values of the age and serum chemistry of the outpatients and admitted patients.

Parameter (median, IQR)	Outpatients	Admitted patients	P
Age, months	60.0 (27.0, 96.0)	36.0 (6.25, 84.0)	0.000
Sodium, mmol/L	137.0 (132.0, 143.0)	135 (130.0, 140.0)	0.000
Potassium, mmol/L	4.2 (3.7, 4.7)	4.0 (3.5, 4.6)	0.002
Chloride, mmol/L	98.0 (94.0, 104.0)	96.0 (92.0, 102.0)	0.002
Bicarbonate, mmol/L	21.0 (19.0, 23.0)	20.0 (18.0, 23.0)	0.000
Urea, mmol/L	3.4 (2.6, 4.6)	4.0 (2.7, 6.3)	0.000
Creatinine, μmol/L	47.0 (35.0, 60.0)	54.0 (39.3, 83.0)	0.000

IQR–Interquartile range.

### Derangements in serum electrolytes and kidney function

Derangements in serum electrolytes were found in 1501 (78.6%) results. About a third each had one (n = 512, 34.1%) or two (n = 499, 33.2%) deranged electrolytes, a quarter (n = 388, 25.8%) had three while 102 children (6.8%) had derangements in all four electrolytes (Table 3). Sodium abnormalities were seen in 52.7% of results, chloride abnormalities in 41.9%, bicarbonate abnormalities in 41.2% and potassium abnormalities in 25.9%. Azotaemia was found in 382 of 1905 patients (20.1%), with infants accounting for almost half this number (42.7%). Serum creatinine levels were at least 1.5 times higher than the upper limit of normal for age in 399 children (24.7%), 239 of them (59.9%) being children admitted for non-surgical reasons. Of the children with elevated serum creatinine, 96 (24.1%) were in the severe category.

**Table 3:** Serum chemistry abnormalities in various age categories.

	Age category n (%)				
**Biochemical parameter**	**Infant**	**Under-five**	**Older child**	**Total**	**P**
**Sodium**					0.197
Normal	257 (49.5)	319 (45.2)	327 (47.9)	903 (47.3)	
Hyponatraemia	194 (37.4)	307 (43.5)	283 (41.4)	784 (41.1)	
• Severe hyponatraemia	32 (16.5)	44 (14.3)	31 (11.0)	107 (13.6)	
Hypernatraemia	68 (13.1)	80 (11.3)	73 (10.7)	221 (11.6)	
Total	519	706	683	1908	
**Potassium**					0.000
Normal	360 (70.3)	540 (77.0)	504 (73.8)	1404 (74.0)	
Hypokalaemia	81 (15.8)	130 (18.5)	147 (21.5)	358 (18.9)	
• Severe hypokalaemia	9 (11.1)	16 (12.3)	21 (14.3)	46 (12.8)	
Hyperkalaemia	71 (13.9)	31 (4.4)	32 (4.7)	134 (7.1)	
Total	512	701	683	1896	
**Chloride**					0.006
Normal	315 (60.7)	400 (56.7)	392 (57.4)	1107 (58.0)	
Hypochloraemia	148 (28.5)	257 (36.4)	235 (34.4)	640 (33.5)	
Hyperchloraemia	56 (10.8)	49 (6.9)	56 (8.2)	161 (8.4)	
Total	519	706	683	1908	
**Bicarbonate**					0.010
Normal	249 (48.5)	433 (61.4)	433 (63.6)	1115 (58.7)	
Acidosis	250 (48.7)	240 (34.0)	217 (31.9)	707 (37.2)	
Alkalosis	14 (2.7)	32 (4.5)	31 (4.5)	77 (4.1)	
Total	513	705	681	1899	
**Urea**					0.000
Normal	357 (68.7)	608 (86.4)	558 (81.9)	1523 (79.9)	
Azotaemia	163 (31.3)	96 (13.6)	123 (18.1)	382 (20.1)	
Total	520	704	681	1905	
**Creatinine**					0.000
Normal, n (%)	287 (69.2)	429 (70.8)	503 (84.2)	1219 (75.3)	
Mild derangement, n (%)	50 (12.0)	79 (13.0)	40 (6.7)	169 (10.4)	
Moderate derangement, n (%)	43 (10.4)	65 (10.7)	26 (4.4)	134 (8.3)	
Severe derangement, n (%)	35 (8.4)	33 (5.4)	28 (4.7)	96 (5.9)	
Total	415	606	597	1618	

Derangements in potassium and bicarbonate were significantly more common in infants, with higher proportions of hypernatraemia, hyperkalaemia, hyperchloraemia and metabolic acidosis than other age categories (p < 0.05 in all cases except for hypernatraemia) (Table 3).

Electrolyte derangements occurred in 80.8% of infants, 78.0% of children under five years and 77.6% of older children (χ^2^ = 1.992, p = 0.369). There was no significant association between age category and the number (χ^2^ = 5.222, p = 0.734) of deranged electrolytes. Electrolyte derangements were more common in children presenting to medical wards and units compared to those from surgical wards and units(77.3% vs 22.7%;χ^2^ = 5.276, p = 0.022) and children who were admitted compared to outpatients (68.1% vs 31.9%;χ^2^ = 22.843, p = 0.000). Among the admitted cases, 846 (72.9%) of the results from the medical wards had deranged electrolytes compared to 176 (51.6%) of the results from the surgical wards (χ^2^ = 23.641, p = 0.000). Among medical cases, the more derangements a patient had, the more likely he/she was to be admitted (χ^2^ = 38.866, p = 0.000). This was not so for surgical cases (χ^2^ = 1.643, p = 0.801).

The association between age category and presence of derangement was significant for both urea (χ^2^ = 61.133, p = 0.000) and creatinine (χ^2^ = 46.824, p = 0.000). Almost a quarter (24.1%) of the derangements in serum creatinine was in the severe category and more common in infants.

There were significantly more derangements in admitted patientscomparedto outpatients for both serum urea and creatinine. For inpatients, those on medical wards had a significantly higher proportion of derangement in serum urea than those admitted to the surgical wards. This picture was not seen with serum creatinine in which the proportions of derangements were similar. One hundred and eight outpatients (17.8%) had deranged serum creatinine (Table 4).

**Table 4:** Proportions of derangements in urea and creatinine in different patient categories.

Patient category	Source	Deranged urea	Deranged creatinine
Inpatient	Medical wards	28.0% (284 of 1015)	28.8% (239 of 829)
	Surgical wards	9.6% (22 of 230)	28.7% (52 of 181)
	Total	24.6% (306 of 1245)	28.8% (291 of 1010)
Outpatient	Medical clinics	11.0% (48 of 436)	17.8% (71 of 400)
	Surgical clinics	12.5% (28 of 224)	17.8% (37 of 208)
	Total	11.5% (76 of 660)	17.8% (108 of 608)

χ^2^ (inpatient vs outpatient urea) = 45.914, p = 0.000.

χ^2^ (inpatient vs outpatient creatinine) = 24.937, p = 0.000.

## Discussion

Of all the children for whom serum chemistry was requested on presentation to the hospital, derangements in serum electrolytes were identified in over three-quarters (78.6%), while derangement in kidney function, as evidenced by elevated serum creatinine to at least 1.5 times the upper limit of normal, was found in almost a quarter (24.7%) of patients.

Electrolyte derangements in children have been reported to various extents in different parts of the world [1, 2, 18, 19, 22–24], with some of these studies performed in children with specific disease conditions. Okposio et al. [18] and Onyiriuka et al. [19] from the southern part of Nigeria reported much higher figures of 89.2% and 91.9% in children < 5 years of age presenting with dehydration related to acute diarrhoeal disease, which is not unexpected [25]. In a recent study by Naseem et al., the prevalence of electrolyte abnormality (assessing 5 electrolytes) amongst children admitted to the PICU of a public sector hospital in Pakistan was reported to be 84% [2]. A prospective, multicentre study in the United States over two decades ago reported electrolyte abnormalities in 68% of children presenting to the emergency departments who had electrolyte panels requested, a quarter of them being clinically significant [22]. Our finding is much higher than the 45.1% reported by Elala et al. in a recent study carried out in a large teaching hospital in Ethiopia [1]. Children in that study were drawn from both the paediatric emergency and intensive care units but only three electrolytes (sodium, potassium and calcium) were studied. A figure of 48% was obtained in a study by Wathen et al. [26] in the USA in children less than 9 years of age presenting to the emergency department and requiring intravenous rehydration. Panda et al. [27] and Agarwal et al. [28] reported figures of 44.3% and 60% respectively in critically ill children admitted to tertiary hospitals in India. Panda et al. studied only two electrolytes (sodium and potassium) while Agarwal et al. used a much wider range for serum sodium as normal and excluded children with gastroenteritis. These reasons could probably explain why their figures were lower than ours. An earlier prospective study on 305 critically ill children in India reported a much lower prevalence of electrolyte derangement of 32% but only two electrolytes were considered in that study, and they also excluded children with diarrhoea and dehydration [24]. We were unable to comment on the prevalence of diarrhoea and vomiting in our cohort as we did not obtain clinical correlates.

Sodium abnormalities were seen in over half of the results of the studied population, the most common electrolyte abnormality being hyponatraemia in 41.1%. This is similar to previous reports from different regions of the world [1, 18, 19, 23, 27, 28] and was expected considering the high prevalence of malaria and gastroenteritis among children in Nigeria [29–31] which have been associated with hyponatraemia [23]. This highlights the need to actively investigate and manage hyponatraemia, which has previously been found to be an independent predictor of mortality [32]. Hypernatraemia was more commonin infants in this study similar to previous reports [1, 33] Younger children tend to lose relatively more electrolyte-free water than adults, making them prone to hypernatraemia [34]. This is of particular importance as hypernatraemia has been associated with higher odds of mortality even when compared to hyponatraemia [27].

Metabolic acidosis was the second most common electrolyte abnormality seen in this study with a prevalence of 37.2%. This was lower than the 54% reported by Rothrock et al. [22] (using broader values from different health facilities) but higher than the 29% reported by Wathen et al. [26] using stricter values to define metabolic acidosis). Onyiriuka et al. [19], while not reporting specific figures for metabolic acidosis, reported that it was significantly associated with mortality when combined with other electrolyte abnormalities in children with diarrhoea. In other studies carried out in public tertiary hospitals in the south-southern part of Nigeria, Uka et al. [20] found metabolic acidosis to be the most common electrolyte abnormality (63.6%), while it was the second most common electrolyte abnormality in the study by Okposio et al. [18] with a prevalence of 59.5%. These very high figures could be explained by the fact that the studies were carried out in children who were admitted with dehydration following acute diarrhoeal disease.

Hypochloraemia was the third most common electrolyte abnormality occurring in a third of study patients. This was comparable to the 34.1% reported by Uka et al. in children who were admitted for diarrhoea [20]. Many studies have not reported chloride [35]. Onyiriuka et al. found relatively low mean values of serum chloride in their study population but did not mention the proportion with deranged chloride values [19]. Rothrock et al. found a prevalence of 8% in their study but no explanation was given for this low figure [22].

Potassium abnormalities are well described in sick children. This study found potassium abnormalities in a quarter of the results, three-quarters of which were hypokalaemia. This is similar to findings from several studies [1, 2, 18, 23, 27, 28] Rothrock et al. [22] reported hyperkalaemia to be more common than hypokalaemia, with no clear reasons for this finding. Fifty-three percent of the children with hyperkalaemia in our study were infants, similar to findings by Elala et al. who reported approximately three-quarters of cases being in infants [1]. Potassium abnormalities, particularly hyperkalaemia, have been associated with increased risk of mortality [1, 27, 28]. While the clinical effects of hypokalaemia are largely neuromuscular, cardiac arrhythmias also occur [36].

While this study did not assess mortality among children with electrolyte derangements, the association between the presence and number of any electrolyte derangements with mortality has been reported by different authors [1, 2, 19, 28].

In terms of kidney function, although most previous studies in Nigeria have focussed on hospitalized children [14, 37, 38], the finding that between 10–20% of outpatients had deranged urea and/or creatinine suggests that a substantial proportion of children who present to the hospital without being admitted may have derangements in kidney function. It also implies that it may have been unnecessary to order serum chemistry in some patients. However, outcomes were not reviewed and this requires further clinical investigation.

The significantly larger proportion of inpatients with deranged kidney function compared with outpatients is not surprising, and has been reported previously [14, 17, 39]. Chami et al. [40] in Tanzania found a lower figure of 16.2% in their prospective study which involved only paediatric medical admissions (excluding children < 2 years of age). Another study by Kimaro et al. [41] found a higher figure of 31.4% in children with sickle cell disease. Children with chronic conditions like sickle cell disease (SCD) and human immunodeficiency virus (HIV) infection have higher rates of kidney dysfunction than the general population [41, 42]. We were unable to report on comorbidities, given the retrospective study methodology.

Some of the discrepancies between our results and previous reports from Nigeria [14, 37, 38, 43, 44] may be a result of different definitions of kidney dysfunction. Comparison of results of studies which used different definitions is known to be difficult especially from an epidemiologic standpoint [45]. Our study used proportions of rise from the ULNs of creatinine while Ademola et al. [37] in a prospective study, used baseline serum creatinine values back-calculated from age-based estimated GFR values (calculated using the Schwartz formula) and defined AKI based on the degree of rise from the baseline value. Esezobor et al. [14] and Anigilaje et al. [44] defined AKI using the paediatric Risk, Injury, Failure, Loss and End-Stage (RIFLE) criteria and found a higher prevalence of AKI than two other similarly retrospective studies [38, 43], which defined AKI based on degree of decline in renal function manifested by rising plasma creatinine levels and reduction in urine output. Our study may thus have underestimated the burden of kidney dysfunction to some degree. The results however suggest that children admitted to hospital should be evaluated for symptoms and signs of kidney disease, and that these should be properly investigated with a view to preventing or slowing disease progression, particularly in settings like ours where resources for long term management are limited.

This study also showed that kidney dysfunction was more frequent in infants. Almost half of the children with azotaemia were infants. Measurement of kidney function in infants is more challenging than in older children with infants exhibiting substantial developmental changes in kidney function [46], with a wide range of normal values for serum creatinine [47] and GFR during the period [48]. Infants are also at higher risk for the development of malaria, sepsis and gastroenteritis (the common causes of non-structural acute kidney injury in this environment) [49]. Mortality in this age group is also higher [50].

This study was limited by the retrospective design, which precluded analysis of admission diagnoses, associated clinical findings and clinical outcome correlates. Kidney dysfunction, as determined by the degree of derangements in serum creatinine, could not be categorized as either acute or chronic and serum eGFR could not be estimated as we could not retrieve the heights/lengths of patients. The possible association between presenting symptoms and socioeconomic conditions with electrolyte derangements and their mechanisms could not be assessed as the information was not available. Furthermore, although the patients who present to this facility come from several states within and outside the northwest region of the country, the fact that the study was from a single-center limits its generalizability. The period of time covered by the study included much of the dry season and only the beginning of the rainy season in Kano and a seasonal pattern for the occurrence of kidney dysfunction could not be deduced as there was no available clinical information in the laboratory records.

As outpatient serum chemistry results in AKTH are only available for collection after 48 hours following specimen submission, it is unlikely that physicians reviewed and acted on the outpatient results raising questions regarding the usefulness of carrying out outpatient serum chemistry tests, when they were unlikely to impact on acute patient management. Although this may be a local issue specific to this institution, it is important for the hospital to institute a system in which serum chemistry results are made available to, or can be accessed by the ordering physician earlier than 24 hours so that the laboratory results can be utilized for acute patient care and possibly monitoring. In the context of limited resources, it may be possible to make more judicious use of the limited resources for laboratory testing and diagnosis of kidney disease. Knowledge of local epidemiology could help drive efforts to improve earlier detection and management. Further prospective clinical studies are recommended in this region to determine the association between derangements and clinical predictors and outcomes.

## Conclusion

This study highlighted the burden of electrolyte derangement and kidney dysfunction in children who had a blood test on presentation to hospital. With the proportion of unreviewed but deranged results found in tests done in outpatients, measures to ensure prompt availability of results should be instituted and the utility of routine testing of serum electrolytes, urea and creatinine in all children who present to hospital for the first time should be reassessed. A study which highlights the clinical presentation and possible admission diagnoses of patients is necessary considering the magnitude of electrolyte derangements found in this study.

## Supporting information

S1 Appendix Normal ranges used for serum electrolytes and terminologies used for deranged values [21, 51]. (DOCX)

S1 Questionnaire Inclusivity in global research. (DOCX)
