# Supplementary material for: Burden and severity of deranged electrolytes and kidney function in children seen in a tertiary hospital in Kano, northern Nigeria
Source: PLoS One. 2023 Mar 17;18(3):e0283220. doi: 10.1371/journal.pone.0283220 (PMC10022757; doi:10.1371/journal.pone.0283220)
Supplement: S1 Appendix — (DOCX) [file pone.0283220.s001.docx]

**Appendix: Normal ranges used for serum electrolytes and terminologies used for deranged values[21,51]**

| **Serum Electrolyte** | **Normal range** | **Terminologies used for deranged values** |
| --- | --- | --- |
| Sodium (mmol/L) | 135 - 145 | < 135 - hyponatraemia  <125 - severe hyponatraemia  >145 –hypernatraemia |
| Potassium (mmol/L) | 3.5 – 5.4 | < 3.5 - hypokalaemia  <2.5 - severe hypokalaemia  >5.4 – hyperkalaemia |
| Chloride (mmol/L) | 95 – 108 | <95 - hypochloraemia  >108 –hyperchloraemia |
| Bicarbonate (mmol/L) | 20 – 28 | <20 - metabolic acidosis  >28 - metabolic alkalosis |
| Urea (mmol/L)  ≤ 12 months of age  12 - 120 months  > 120 months | 1.2 – 5.8  3.2 – 7.6  2.6 – 6.5 | >5.8 – azotaemia  >7.6 – azotaemia  >6.5 – azotaemia |
| Creatinine (µmol/L)  <12 months  12 – 48 months  48 – 84 months  84 – 144 months  144 – 180 months | 28 – 47  34 – 48  39 – 57  46 – 61  50 - 71 | Mild derangement = 1.5 – 1.9 x ULN  Moderate derangement = 2.0 – 2.9 x ULN  Severe derangement = ≥3 x ULN |

ULN – Upper limit of normal
